# Olov Oscarsson (1931–1996) of Lund University, a Pioneer in Cerebellar Neurobiology

**DOI:** 10.1007/s12311-023-01515-7

**Published:** 2023-01-24

**Authors:** Lazaros C. Triarhou, Mario Manto

**Affiliations:** 1 Department of Psychology, Sector of Cognitive, Behavioral and Brain Sciences, Faculty of Philosophy, Aristotelian University, University Campus, Thessaloniki, 54124 Greece; 2 Unité Des Ataxies Cérébelleuses, CHU-Charleroi, Charleroi, Belgium; 3 Service Des Neurosciences, University of Mons, Mons, Belgium

**Keywords:** Cerebellar afferent innervation, Cerebellar longitudinal somatotopy, Cerebellar microzones, Cerebellar corticonuclear microcomplexes

## Abstract

The present *Cerebellar Classic* highlights the experimental work of the Swedish neurophysiologist Olov Oscarsson (1931–1996) on the afferent innervation of the cerebellum by axons emanating from neurons in the spinal cord and the inferior olive. Historically, the schemes of cerebellar division had been principally based on the external morphology of lobules and fissures. However, the macroscopic anatomical division of the cerebellum does not coincide with its pattern of functional organization. By defining a system of longitudinal somatotopy, Oscarsson contributed to the much needed plan of cerebellar division that correlates experimental information on axonal connections with physiology. His contribution has ultimately led to the currently accepted microzonal modular scheme of cerebellar corticonuclear microcomplexes.

Our current understanding of the cerebellar operational unit rests with the sagittal microzone, a concept commensurate with the cerebral cortical column [1]. Rather than relying on cytoarchitectonics, which is the case for the cerebral cortex, the organizing principle in the cerebellum is based on longitudinal subdivisions regarding neurogenetic attributes, molecular identities, and afferent and efferent connections, whereby each microzone conceivably regulates a particular functional mechanism. The cerebellar microzones reach a maximum width of 200 μm and are oriented perpendicularly to the long axis of the folia and the parallel fibers; their orientation corresponds to the sagittal plane where the folia are arranged transversely [1]. The idea of the microzones as fundamental cerebellar processing units has been advanced by the excellent work of the Swedish neurophysiologist Olov Oscarsson (1931–1996) at Lund University (Fig. 1). His contribution followed earlier anatomical work that had shown the existence of at least five differentiations on each side of the midline in the anterior cerebellar lobe of ferrets [2], and a longitudinal organization with three sagittal subdivisions in the hemivermis and at least two sagittal subdivisions in the pars intermedia of the developing cerebellum of cetaceans and rodents [3, 4].

**Fig. 1:**
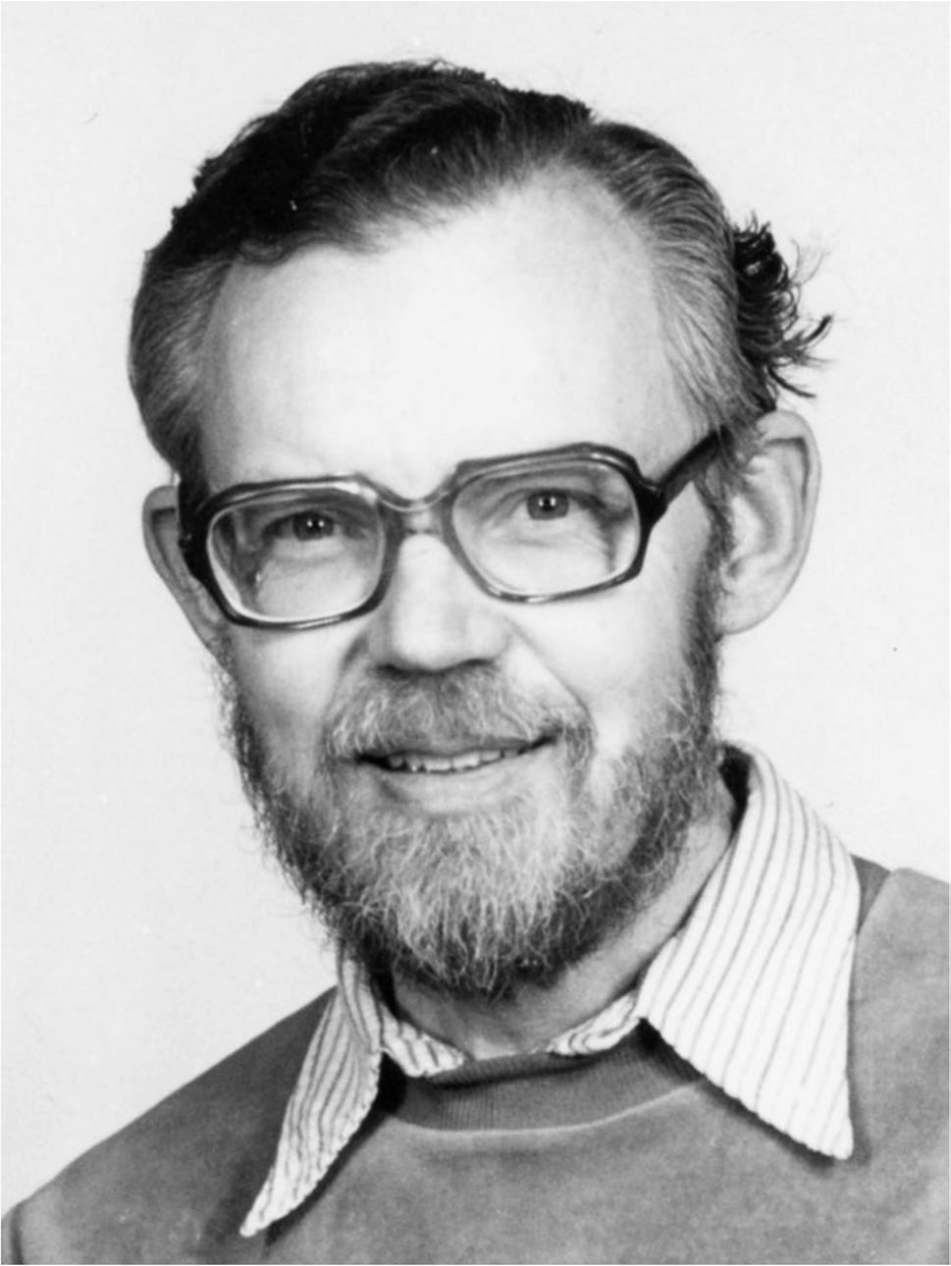
Professor Olof Oscarsson at the Physiological Institute of Lund University. Photo by Olle Hammar. Credit: Öppet Bildarkiv, Sydsvenska Medicinhistoriska Sällskapet, Lund. https://www.medicinhistoriskasyd.se/SMHS_bilder/displayimage.php?pid=12643

Oscarsson showed that climbing fiber potentials, upon stimulation of peripheral nerves, and Purkinje cell complex spikes observe a discrete parasagittal zone pattern [5, 6]. The careful physiological studies of Oscarsson and his collaborators on the spino-olivocerebellar pathways were particularly important for understanding the theme of the somatotopic pattern in the distribution of spinal input to the inferior olive, and the projections from the relevant parts of the inferior olive to the cerebellum [7]. The projection patterns formed by five spinal paths terminating as climbing fibers in the anterior lobe of the feline cerebellum revealed that the pars intermedia consists of at least four narrow sagittal zones and the hemivermis of at least two sagittal zones [5]. Different parts of the inferior olive convey information from one or several specific spino-olivocerebellar pathways that can be readily identified with electrophysiological mapping techniques [8, 9]. Oscarsson and Ingmar Rosén also discovered another effective spino-olivary pathway that ascends up the dorsal columns to the dorsal column nuclei and presumably reaches the contralateral accessory olive via collaterals from the medial lemniscus [10, 11].

This year marks the semicentennial of Oscarsson’s *Cerebellar Classic* [12], in which he reviewed the several groups of pathways ascending through different tracts of the spinal cord and mediating spinal impulses to the cerebellum via the inferior olive, distinguished on the basis of differences in receptive fields and the latency of the cerebellar responses to peripheral stimulation, among other criteria.

There are 72 papers authored or co-authored by Oscarsson in PubMed, published from 1953 to 1987. He collaborated with neuroscientists of international renown, including historical figures such as Sir John C. Eccles (1903–1997), Anders Lundberg (1920–2009), Platon Grigorevich Kostyuk (1924–2010), Tatsunosuke Araki (1926–1985), and Masao Ito (1928–2018); other collaborators included Gerald Andersson, Carl Fredrik Ekerot, Gunnar Grant, Birgitta Holmqvist, Bengt Larson, Rosén, Jens Schouenborg, Bengt Sjölund, and Nils Uddenberg.

Jan Voogd [2] demonstrated the anatomical organization of the inputs to the cerebellar cortex into longitudinal zones for the pattern of olivocerebellar projections, and Oscarsson provided electrophysiological evidence for the pattern of spinocerebellar inputs [6]. Oscarsson discovered that spinocerebellar tracts target the anterior lobe and lobule VIII or spinocerebellum, without projections to the lateral cerebellum or neocerebellum [1]. He suggested a microzonal structure of the cerebellar cortex as an elemental structural–functional unit with its specific efferent and afferent connections [1, 13, 14].

Oscarsson and Voogd met in March 1969 during the First International Symposium on the Neurobiology of Cerebellar Evolution and Development that was organized by Rodolfo Llinás in Chicago, where Voogd presented his anatomical ideas on the longitudinal zonal organization of the cerebellum [15, 16]. Oscarsson had already published on spino-olivary climbing fiber systems in the preceding years [17, 18]. At the 1969 Symposium, he summarized his observations on the climbing fiber system and the functional division of the anterior cerebellar lobe of the cat into multiple sagittal zones and formulated a working hypothesis on the relation of such zones to the function of the cerebellar cortex. Each sagittal zone would receive information—via spinocerebellar and cuneocerebellar tracts activated from proprioceptors and exteroceptors in the periphery and terminating as mossy fibers to the entire width of the anterior lobe—related to a different control mechanism, and its primary function would be to correct motor acts handled by that mechanism [6]. Oscarsson and his collaborators analyzed the ventral [18, 19], dorsal [14, 20], dorsolateral [21], and lateral spino-olivocerebellar pathways [22], and produced a map of the somatotopic organization of such longitudinal zones [18].

Voogd and Oscarsson realized that they were studying identical systems, and in his later studies, Oscarsson adopted Voogd’s nomenclature for the cerebellar zones [16]. In his presentation, Oscarsson also mentioned the relation of the sagittal zones to the projection areas of climbing fiber paths from the cerebral cortex and the mesencephalon. In the discussion that followed [6], Voogd further commented on the descending pathway to the inferior olive via the central and medial tegmental tracts originating in the red nucleus and the nucleus of Darkschewitsch, areas that in turn receive afferent innervation from the cerebellum, and emphasized the role of the inferior olive not only as a link from the periphery to the cerebellum, but also as a recurrent pathway originating in and returning to the cerebellum.

Voogd praises Oscarsson’s work as being consistently accurate, reliable, and devoid of speculations, characteristics typical of the group of neurophysiologists at Lund University. Furthermore, Voogd has described Oscarsson as a modest person, albeit assertive when it came to his research, who inspired his students and greatly extended our knowledge of cerebellar microzones [16]. In a joint study with Andersson, Oscarsson described them in the B zone of the cat cerebellum—in other words, narrow longitudinal strips of Purkinje cells receiving climbing fibers that share the same peripheral receptive field [23].

In his initial collaboration with Lundberg and Eccles, Oscarsson conducted a series of experiments in cats in order to identify and characterize the ascending spinocerebellar pathways. Actually, Oscarsson’s focus on the cells of origin of the spinocerebellar tracts was a key factor that led Eccles to move into the complex and challenging problems at higher levels of the central nervous system, after spending a decade on intracellular recordings of the spinal cord [24]. In the context of the long spinal pathway that conveys inhibitory signals, a monosynaptic inhibition is induced in some ventral spinocerebellar tract neurons in the spinal cord [25].

Ventral spinocerebellar tract fibers are often spontaneously active, particularly in unanesthetized animals [26]. These fibers, and their forelimb homolog, the rostral spinocerebellar tract, are more restricted than the dorsal spinocerebellar tract with regard to modality [27]. They relay information almost exclusively from Golgi tendon organs (I*b*), but they are wider with regard to the muscle from which the muscle afferents converge onto any one fiber [25, 26, 28]. These tracts seem to signal stages of muscle contraction and the interaction between contraction and resistance to the movement of the entire limb [27].

Oscarsson studied both the spinocerebellar and spino-olivocerebellar pathways [8, 28]. By transecting the spinal cord, he recorded from single Purkinje cells, analyzed the climbing fiber responses, and reported a *longitudinal somatotopy* of climbing fiber projections into the anterior cerebellar lobe via the ventral tract [18]. This was in contrast to the transverse somatotopic organization of afferent projections previously suggested by Adrian [29] and Snider and Stowell [30], among others [31].

The spinoreticulocerebellar pathway conveys exteroceptive information from the forelimb and hindlimb of either side to the lateral reticular nucleus, and thence via the inferior cerebellar peduncle to the ipsilateral anterior cerebellar lobe [10, 32, 33]. As this pathway only gives poor somatotopic localization, Oscarsson [11] suggested that it conveys information about the levels of interneuronal activity in the spinal cord, contrary to the somatotopic projection of exteroceptive information [29, 30]. Moreover, in an interpretation consistent with that of Eccles et al. [27], Oscarsson [18] suggested that the transverse organization of Snider and Stowell [30] and Combs [34] actually represents climbing fiber projections via the dorsolateral and dorsal spino-olivocerebellar pathways.

Oscarsson’s observations [28] indicated that the fibers of the dorsal spinocerebellar tract influence a small group of cerebellar cortical neurons, whereas ventral and rostral spinocerebellar fibers influence cerebellar cortical neurons scattered over a wide area, features attributed to differences between the mossy fibers of such tracts with regard to the profuseness of their branching [35].

There is electrophysiological evidence for the wide dispersion of mossy fiber collaterals into the cerebellar cortex. Specifically, single fibers in the ventral and rostral spinocerebellar tracts could be antidromically activated by weak stimulation over fairly widespread domains of the anterior lobe of the cerebellum, where there is a much smaller excitatory area for dorsal spinocerebellar fibers [32, 36, 37].

The rostral spinocerebellar tract is uncrossed in the spinal cord and enters the cerebellum via both the superior and inferior cerebellar peduncles on the ipsilateral side. It is distributed to both the forelimb and hindlimb areas of the anterior cerebellar lobe, and it is concerned with the correlation of patterns of muscle contraction in both limbs [38].

Tract fibers are engaged in forwarding information from group I*a* or I*b* receptors of a synergic group of muscles [39, 40]. The origin of the ventral spinocerebellar tract in the cat was investigated by means of intracellular recordings obtained after monosynaptic excitation by Golgi tendon organ (I*b*) afferents and antidromic activation from the contralateral side. It was found to lie in a column of neurons in the lateral part of the intermediate zone and the dorsolateral part of the ventral horn at the upper *L*_5_ or lower *L*_4_ spinal segments, with some cells also located in the adjacent part of the dorsal horn [41].

Physiologically, there are differences between the dorsal spinocerebellar and cuneocerebellar tracts [28, 35, 42, 43]. These pathways convey impulses from muscle spindles and tendon organs as well as from cutaneous receptors, whereas the ventral and rostral spinocerebellar tracts appear to be devoted to the transmission of proprioceptive impulses [28, 35]. Stimulation of the superficial radial nerve evoked, after a latency, a brief burst discharge of a neuron in the granule cell layer, probably via the cuneocerebellar tract with its mossy fiber terminals. Thus, the cuneocerebellar tract provides a comparable pathway for muscle receptors of the forelimb, and it is distributed to the lateral vermis and the medial part of the intermediate zone of lobule V. On the other hand, the dorsal spinocerebellar tract is fairly sharply restricted to the more rostral lobule IV [27, 42]. The hindlimb projection by the dorsal and ventral spinocerebellar tracts is sharply separated from the forelimb area, as far as this is conveyed over the external cuneocerebellar tract, with the boundary at the border between lobules IV and V [28].

In later somatotopic maps, both direct and indirect pathways were distinguished, suggesting that inputs from the hindlimbs are represented in the medial parasagittal zones of the anterior lobe, whereas inputs from the forelimbs are represented in the lateral parasagittal zones in both the ventral and dorsal spino-olivocerebellar pathways [20, 31].

All ventral spinocerebellar tract neurons receive strong polysynaptic input from ipsilateral *flexor reflex afferents*, defined as myelinated fibers that evoke a flexor reflex in the spinal preparation, and comprising low- and high-threshold cutaneous fibers, group II and III muscle afferents, and high-threshold joint afferents [12]. Components of the flexor reflex afferents can be either inhibitory or excitatory; they exert polysynaptic effects, mediated by a pool of interneurons strongly excited or inhibited by descending tracts. Flexor reflex afferents represent the major input of spinocerebellar tracts, and they are more suitable to monitor the pool of interneurons commanding the motoneurons than to convey accurate peripheral sensory information [44].

Ito [45] extended Oscarsson’s concept of longitudinal somatotopy to that of the corticonuclear microcomplex, which he described in the following terms: the cerebellum consists of numerous functional units, called *cerebellar corticonuclear microcomplexes*. Such microcomplexes comprise four elements: (1) a cerebellar cortical microzone as defined by Oscarsson [46]; (2) a small group of neurons in the vestibular or the cerebellar nuclei which receive inhibitory innervation from Purkinje cells of the microzone; (3) bundles of cerebellar afferent fibers arising from small groups of precerebellar structures and establishing excitatory synapses with neurons of the cerebellar nuclei, as well as mossy fiber terminals to the microzone; and (4) a bundle of climbing fibers in the microzone, originating from a small group of inferior olivary neurons and forming excitatory synapses with neurons of the cerebellar nuclei. While a mossy fiber input is converted to a nuclear output through a microcomplex, the input–output relationship is modifiable due to long-term depression induced in the microzone by error signals conveyed by the climbing fibers. Thus, the microcomplex is an adaptive unit of the cerebellum: Like a computer chip, it is attached to a bodily control system and affords adaptiveness [47].

Ito [45] indicated that each functional corticonuclear complex could compute a certain input–output function, modifiable by error signals conveyed by the climbing fibers. In different situations, the corticonuclear complex might represent a simple adaptable dynamic model. A corticonuclear microcomplex connected within a positive feedback loop could account for integrator tuning in the cerebellum. Alternatively, if located in the forward path, a microcomplex could effect a direct adaptive feedback servo controller [48].

Richard Apps and Martin Garwicz [49] modified the concepts of Oscarsson [1] and Ito [45], such that a strict correspondence would be maintained between input and output in spatially separate microzones. A specific convergence of information to the same region of a cerebellar nucleus arises from multiple similar microzones in the cerebellar cortex.

In the companion commentary, Tom Ruigrok [50] discusses the implications of the pioneering work of Oscarsson, and its impact on current research in cerebellar neurobiology.

## Data Availability

Data sharing not applicable to this article as no datasets were generated or analyzed during the current study.
